# Estimating the impact of COVID-19 self-test availability and modifications in test-strategy on overall test uptake using an experimental vignette study

**DOI:** 10.1038/s41598-024-54988-9

**Published:** 2024-03-11

**Authors:** Colene L. Zomer, Floor Kroese, Jet G. Sanders, Riny Janssen, Marijn de Bruin

**Affiliations:** 1 Corona Behavioural Unit, National Institute of Public Health and the Environment, Bilthoven, The Netherlands; 2 Radboud University Medical Center, Institute of Health Sciences, Nijmegen, The Netherlands; 3 Department of Clinical and Health Psychology, Utrecht University, Utrecht, The Netherlands; 4 Department of Psychological and Behavioural Science, London School of Economics and Political Sciences, London, UK; 5 Centre for Infectious Disease Control, National Institute of Public Health and the Environment, Bilthoven, The Netherlands

**Keywords:** Human behaviour, Public health

## Abstract

To inform future Dutch COVID-19 testing policies we did an experimental vignette study to investigate whether inclusion of the less reliable lateral flow tests (self-tests) would change test-uptake sufficiently to improve population-level test sensitivity. A representative sample (n = 3,270) participated in a 2-by-2 online experiment to evaluate the effects of test-guidelines including self-testing advice (IV1), and the effects of self-test availability (IV2) on expected test uptake (PCR test, self-test or no test) and sensitivity of the overall test strategy (primary outcome). Across four scenarios, changing test advice did not affect expected testing behaviour. Self-test availability, however, increased the timeliness of testing, the number of people testing, and overall test strategy sensitivity. Based on these findings, we recommend that (national) policy facilitates a supply of self-tests at home, for example through free and pro-active distribution of test-kits during a pandemic. This could substantially enhance the chances of timely detecting and isolating patients.

## Introduction

Throughout the pandemic, testing for COVID-19 has been key to monitoring the spread of SARS-CoV-2 and slowing down the spread of the virus through isolation of infected individuals, contact tracing and quarantine. Between July 2020 and May 2022, Dutch citizens with symptoms indicative of COVID-19 were eligible to test for free at test facilities from the Municipal Health Services (MHS) for (primarily) Polymerase chain reaction (PCR) tests. Government institutions have consistently advised citizens with symptoms indicative of a COVID-19 infection to get tested at MHS test facilities. This recommendation mostly has two purposes; (1) PCR tests have high test sensitivity, (2) contact tracing is centralised. Lateral Flow Tests for at-home use (self-tests) became available in the Netherlands in May 2021, and were introduced only for testing in situations for prevention, not in case of symptoms.

Despite these recommendations, the proportion of people self-testing with symptoms slowly but steadily increased and testing at MHS test facilities slowly but steadily decreased^1^. This suggests that an increasing proportion of people preferred (less sensitive) self-testing over testing at the MHS, in contrast to government recommendations. This deviation of citizen testing behaviour from governmental testing guidelines was also observed in the United Kingdom^2,3^. Various modelling studies suggest that turnaround time (i.e., how quickly people test after symptom onset) and frequency of testing are more important for COVID-19 control than test sensitivity^4–6^. Hence, changing from primarily a PCR testing strategy to a strategy that allows for the less sensitive but easier to conduct lateral flow tests, might translate into a higher probability of timely COVID-19 detection. The higher the prevalence of infection, the higher the positive predictive value of self-testing^7^. However, there are no studies that have experimentally examined the impact of such a policy change and other strategies (e.g., easier access to self-tests) on the effectiveness of the test strategy. The current study therefore addresses the following question: if the test policy would incorporate a recommendation to use a self-test in case of symptoms, would an increase in self-testing (lower sensitivity) increase overall test-uptake sufficiently to compensate for a possible decline in PCR-testing (higher sensitivity)?

Why was this transition in preference for self-testing observed in the first place? The practical benefits of self-tests are probably one of the main reasons self-test use increased, in the absence of government guidance. Self-tests are relatively easy to use independently and can provide immediate results, as a positive test result implies infectiousness. This also directly affects the tester as symptomatic people with a negative test result were not required to stay at home. A faster diagnosis would also contribute to the timeliness of contact tracing. In accordance with this line of thought, the WHO advised that self-testing should be offered in addition to professionally administered testing services^8^. Given these advantages, it is plausible that more people would test when self-testing is recommended in case of symptoms in addition to PCR testing. This could potentially also lead to people testing more promptly after symptom presentation, and more willingness to repeatedly test when regularly experiencing symptoms.

Besides government recommendations on what test to use, having self-tests readily available—as opposed to having to purchase them when symptomatic (which could also be a moment of increased risk of transmission)—could be another important factor influencing test uptake. Quasi-experimental research has shown that testing at MHS test facilities increases if the distance to test facilities decreases^9^. Other experimental evidence shows that willingness to use self-tests decreases when their costs increase^10^ but also that availability of tests influences isolation intention^11^. In the Netherlands, self-tests are widely available at supermarkets and pharmacies for approximately 3 euros per unit, whereas in countries such as the United Kingdom people could order self-tests free online. We were interested in examining the potential impact of people having self-tests available at home when experiencing symptoms as opposed to having to purchase them or asking someone else to do so. This could provide valuable insights for advising the government to pro-actively distribute free self-test supplies to Dutch citizens.

Given the impossibility of carrying out this research in a real-life randomized trial due to the time-sensitivity of the policy change and practical reasons, we decided to conduct a vignette study on the role of self-tests in close collaboration with epidemiologists and virologists with expertise in COVID-19 testing to advise the Dutch Outbreak Management Team and government. An experimental vignette allows researchers to manipulate independent variables and creates insight into causal relationships which made it a good design for studying test expectations^12^.

In this study we used an online, 2-by-2 vignette to investigate two hypotheses: first, that advising symptomatic people to either self-test or test at an MHS test facility, as opposed to only test at an MHS test facility, increases the probability of detecting positive cases, through an increase in testing and a decrease of time to testing. Second, that the availability of self-tests at home as opposed to having to purchase them, increases the probability of detecting positive cases, through an increase in testing and a testing more promptly after symptom onset. Additionally we explored if the probability of detecting positive cases would decline over time, as people were expected to test less frequently after consecutive episodes of symptoms, and if government advice and the availability of self-tests at home could counter this decline.

All scenarios were carefully designed by following the best practice recommendations to enhance accuracy and experimental realism^12^. Consenting participants were each presented with four episodes of symptoms over four consecutive months in the near future. The type of symptoms in each scenario, ranging from one to multiple symptoms, were based on their actual distribution in the population. Participants were first prompted to depict their typical activities on the relevant weekday in an open text box, which, in the vignettes, featured the initial symptoms occurring on a Tuesday. This step aimed to enhance realism and increase immersion. Next, they were asked to indicate whether—given their symptoms—they expected to not test, go to an MHS test facility or use a self-test that day. If participants did not select the MHS test facility (the optimal test), they were told their symptoms persisted for another 2 days and were asked again what test choice they expected to make. We assessed participants behavioural expectations, as this should reflect behavioural intentions (I plan to do x) adjusted for other influential factors such as past behaviour or specific circumstances on that day. Some studies suggest that behavioural expectations are better predictors of behaviour than behavioural intentions, although the evidence is mixed^13–17^.

To test the hypotheses, respondents were randomly allocated to scenarios describing the testing advice: (1) ‘When you have corona-related symptoms, get tested at an MHS test facility’ (current government recommendation) or the adjusted advice: ‘When you have corona-related symptoms, get tested at an MHS test facility. If this is not possible, use a self-test’; and testing availability: (2) Having self-tests available at home versus not having a supply at home, when COVID-19 symptoms present. The primary outcome was overall strategy sensitivity: an average COVID-19 test sensitivity score based on participants’ test choices on day 1 and by day 3 of symptoms across all four scenarios, taking into account differences in self-test versus PCR tests detection probability (sensitivity)^7,18^ and considering that viral load is highest at the time of symptom onset^19,20^. Additionally, we examined whether participants opted for testing sooner, whether more people opted for testing, and whether this changed over time.

## Results

### Participant flow

**Figure 1:**
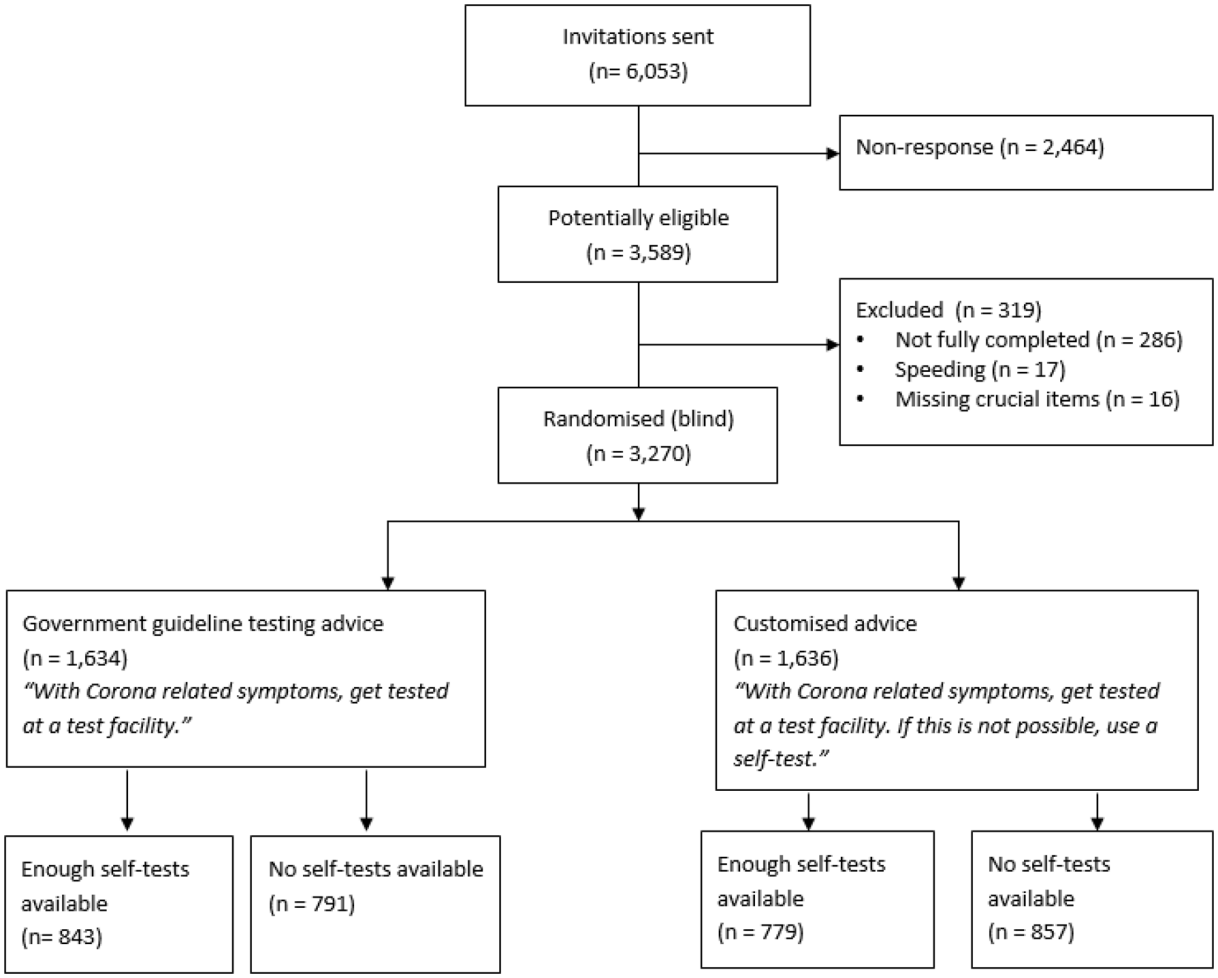
Participant flow diagram.

Of the 6,053 participants who were approached for participation and were selected by an online research panel on demographic representativeness, 3,589 consented. Of those, 319 were excluded due to: fast completion (< 4 min; n = 17), not finishing the questionnaire (n = 286) and not answering crucial questions (n = 16) as per standard protocol from the data collection agency (not described in pre-registration). See Fig. 1 for participant flow diagram*.*

### Descriptive statistics

From a total of 3,270 participants, 50.8% were female. Their median age was 53.3 (standard deviation = 17.3) years and 40.2% was highly educated. 79.5% said to have a Dutch background and 20.2% said to have a migration background (7.7% non-western and 12.5% western). Respondents reported to live alone (29.8%), with a partner (39.2%) or with a partner and children (20.5%). See Supplementary Materials (S1) for more details on demographics.

Across scenarios, respondents assigned to experience multiple symptoms (coughing, sneezing and a slight fever) relative to those assigned to experience one mild symptom indicated that they would test more often (90.3% vs. 74.9%), would do so sooner (77.3% vs. 53.5% immediately), and preferred to do so at an MHS test facility (57%) rather than by means of a self-test (19%) or by means of a self-test followed by a test at an MHS test facility (14%) (see Supplementary Materials S2).

By the third day of symptoms (day 1 and day 3 inclusive), respondents on average opted to test in 78.8% across the four episodes of symptoms (46.9% MHS test facility, 37.5% self-test and 15.5% both; 59.5% tested on day 1). Participants willingness to test varied with time (76.8% November, 81.6% December, 79.4% January, 77.3% February), with the highest test rate before the Christmas holidays: a behaviour we had actually observed in December 2020^21^.

### Primary analyses

**Figure 2:**
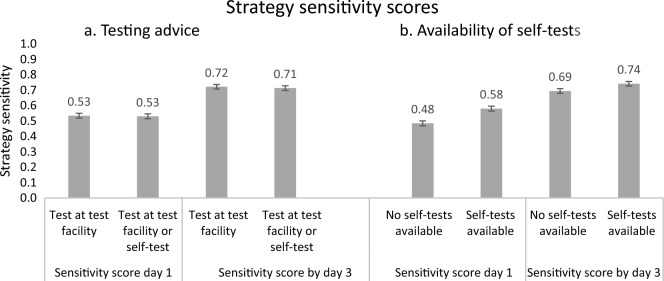
Strategy sensitivity scores, averaged over 4 scenarios, based on choice of testing behaviour with COVID-19 symptoms by testing advice (**a**) and availability of self-tests (**b**) on the first day of symptoms and by day 3 of symptoms.

We calculated the overall strategy sensitivity score (the probability of detecting positive cases) by converting the type of test chosen into the respective sensitivity scores on day 1 and by day 3 of symptoms averaged over all four scenarios, see “Methods” for details. There was no effect of changing the test advice on the overall strategy sensitivity score (F(2, 3265) = 0.43, *p* = 0.654). There was a significant effect of self-test availability at home on the overall strategy sensitivity scores, showing higher sensitivity scores when self-tests were available at home (F(2, 3265) = 38.5, *p* < 0.001, ηp2 = 0.023). This effect was present on day 1 (F(1, 3266) = 67.8, *p* < 0.001, ηp2 = 0.020) and still prevalent by day 3 of symptoms (F(1, 3266) = 19.3, *p* < 0.001, ηp2 = 0.006). There was no significant interaction between test advice and self-test availability (F(2, 3265) = 1.209, *p* = 0.299). Strategy sensitivity scores are shown in Fig. 2.

### Secondary analysis

Next we analysed whether participants opted for testing sooner, and whether more people opted for testing. Test availability had a significant effect on the number of people that expected to test either at an MHS test facility or self-test (F(2, 3265) = 59.42, *p* < 0.001, ηp2 = 0.035). Moreover, when self-test were available at home, people expected to test sooner as more participants indicated to test on Day 1 than participants without self-tests at home (F(1, 3266) = 117.87, *p* < 0.001, ηp2 = 0.035). The significant effects found for testing sooner and more often are driven by the increase in use of self-tests. Also, more participants expected to test (by day 3 of symptoms) with self-tests at home than participants without self-tests at home (F(1, 3266) = 51.03, *p* < 0.001, ηp2 = 0.015). There was no effect of changing the test advice on expected test uptake or time to testing (F(2, 3265) = 0.31, *p* = 0.736).

**Figure 3:**
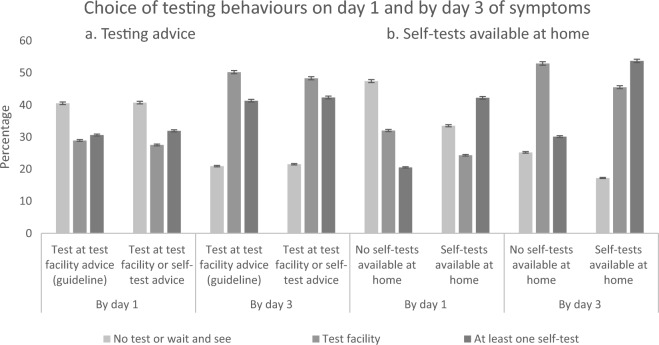
Percentages of selected testing behaviours with COVID-19 symptoms averaged over 4 scenarios by testing advice (**a**) and availability of self-tests (**b**) on day 1 of symptoms and by day 3 of symptoms. By day 3 also contains participants who chose to visit the MHS test facility on day 1. Percentages above 100% represent participants who expected to use a self-test on day 1, and expected to go to the MHS test facility on day 3.

The shift in choice of testing behaviour is shown in Fig. 3. On day 1, expected self-test use doubled resulting in 21.6% difference between groups using self-tests if self-tests were available at home. This comes paired with a decrease of 7.7% of testing at MHS test facilities.

### Consecutive episodes of symptoms over time on strategy sensitivity

**Figure 4:**
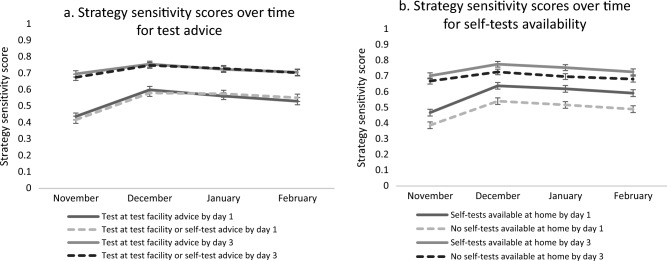
Strategy sensitivity scores for the four scenarios over time, based on choice of testing behaviour with COVID-19 symptoms by testing advice (**a**) and availability of self-tests (**b**) on the first day of symptoms and by day 3 of symptoms.

We expected that strategy sensitivity would decline over time and that people would expect to test less after consecutive episodes of symptoms. However, we also expected that changing the testing advice towards allowing for self-tests in case of symptoms, and having self-tests available at home would counter this decline. Indeed, the consecutive episodes of symptoms over time yielded a significant main effect by day 1 (F(2.99, 9775.38) = 155.23, *p* =  < 0.001, ηp2 = 0.045) and by day 3 of symptoms (F(2.97, 9690.01) = 34.57, *p* ≤ 0.001, ηp2 = 0.01). A Greenhouse–Geisser correction was used due to a violation of sphericity. Pairwise comparisons show that the increase in overall strategy sensitivity was especially large from November to December, after which a small decline set in, yet sensitivity scores remain higher than the start in November (Fig. 4a,b).

The four consecutive episodes of symptoms over time showed a significant interaction with testing advice on strategy sensitivity scores on day 1 (F(3, 3264) = 3.27, *p* = 0.020, η2 = 0.003) suggesting that the effect of testing advice varied over time: strategy sensitivity scores were slightly higher for the current government guideline testing advice (test at an MHS test facility) during the first two months, and then slightly higher for the (self-)testing advice during the last 2 months. This effect was, however minimal, no longer apparent by day 3 (F(3, 3264) = 1.158, *p* = 0.324). No other effects were found on consecutive episodes of symptoms, testing advice and availability of self-tests.

### Robustness check

The primary outcome analyses were repeated using a 10% lower sensitivity score for self-tests (0.7 instead of 0.8 on day 1 and 0.6 instead of 0.7 on day 3), but the conclusions on governmental testing advice and availability remained. No difference was detected for testing advice (F(2, 3265) = 0.50, *p* > 0.05) and availability of self-tests at home still showed a significant difference in sensitivity scores between conditions (F(2, 3265) = 27.9, *p* < 0.001, ηp2 = 0.017). This effect remained strongest on day 1 (F(1, 3266) = 44.08, *p* < 0.001, ηp2 = 0.013) and remained in the maximum strategy sensitivity score on day 3 (F(1, 3266) = 8.68, *p* = 0.003, ηp2 = 0.003).

## Discussion

In this vignette study we found that having self-tests available at home—but not the change in test advice—substantially increased respondents expected test uptake, reduced time to testing, and led to an increase in the overall strategy sensitivity. Following this study the Dutch Outbreak Management Team advised the government to provide free self-tests to all Dutch citizens. An advice that was, however, not followed-up because of market regulation issues. Free self-tests were already provided to people attending food banks, and to students.

In the study, we observed no changes in expected test uptake following a change in testing advice. We note that quite a number of people had already been using self-tests to determine if their symptoms were due to COVID-19. This could explain why we did not observe a difference in people’s expected testing behaviour. It could also be possible that the message was not correctly comprehended by the respondents, as the message was not pre-tested. However, the scenario was under direct consideration by the Dutch Outbreak Management Team during the time of the study. After this study was conducted, Dutch governmental testing advice changed to include a recommendation to self-test when experiencing symptoms. Survey research has since shown that people did report using more self-tests after the advice changed (39% with corona-related symptoms) than before (28.7%)^1^. Nevertheless this increase could have been caused by the increased attention for COVID-19 due to the simultaneous infection peak that occurred upon the introduction of Omicron.

Having self-tests available at home decreased the expected time to testing and substantially increased the number of people who expected to test. Modelling studies have shown that frequent testing, i.e. increasing COVID-19 detection probability, is beneficial for mitigation of COVID-19^4–6^. Furthermore, viral shedding is highest on or just before onset of symptoms^19^, and early transmission could therefore be reduced with sooner testing. This may indicate that providing free self-tests can help mitigate pandemics. Some governments, such as in the UK, have provided free self-tests to the population during the pandemic^22^, which is likely to reduce (financial) barriers to having self-tests at home and thus promote more rapid testing following the onset of symptoms^23^. Evaluation of this widescale testing pointed towards positive effects on public health^24^.This serves as a strong start to improving test uptake, but some groups may need additional provisions as research shows that higher education and above average income is positively associated with motivation to use and order self-tests^25^, and that costs of self-tests are related to intended use^10^. Research on self-reported testing behaviour shows that respondents who find self-tests more expensive report to use self-tests less often with corona-related symptoms. Interestingly, this effect was found across various person characteristics, including those with higher education (as an indicator of financial capacity)^26^. As costs of self-tests could pose a barrier to having self-tests available and using self-tests, providing sufficient self-tests for free would provide a good alternative across the population^27^.

Finally, we did not find evidence of test fatigue, where consecutive episodes of symptoms over time would lead to decline in test uptake^28^. It is possible that the hypothetical nature of our study was not optimal for detecting this. Instead, we have been seeing a modest but steady increase in test uptake over time as self-testing became increasingly popular^29^. Attesting to the validity of our findings, the peak in strategy sensitivity in December was mirrored by self-reported testing data from the corona behavioural unit cohort study and can most probably be explained by December being a traditional time to celebrate with family, when respondents do not want to risk infecting loved ones^1,16^. There are also other indications of the validity of the results from this Vignette study. For example, socio-demographic predictors of expected test behaviour in the Vignette study are similar to those we have seen in our ongoing surveys: women, vaccinated respondents, younger and higher educated respondents test more frequently^1–3^. Also, severity of symptoms in the scenarios was predictive of expected test uptake, in the same way as we have seen in surveys on actual test uptake^1,2,30^. These comparisons support the conclusions from this vignette study, and of the method more generally, as having predictive value.

Strengths of this study are the carefully-designed scenarios using available data (e.g., on type of symptoms and their frequency) and following best-practice guidelines, the multi-disciplinary research team, the nationally representative data and the comparisons that support its validity. Limitations are that this is, ultimately, a vignette study using expected behaviour as an outcome and that conclusions are based on assumptions about the sensitivity of PCR and self-tests. As self-tests may be somewhat less sensitive for Omicron, the variant that rapidly took over in December 2022, than Delta^7^, the variant that circulated between July 2021 and December 2022, this could affect the relevance of the results. However all results were similar when we assumed a 10% lower sensitivity scores for self-tests than Delta sensitivity scores. The self-test availability hypothesis was accidentally categorised under follow-up analysis in the study’s registration. However, as the study was designed and powered with the availability-arm as a main hypothesis in mind, as further described in the pre-registration, we believe we have remained true to our pre-registration.

In conclusion, self-testing for COVID-19 appears to be a relatively low-effort activity that many people agree to doing immediately when symptoms present themselves. Although this study did not provide evidence to support modifications in self-test advice, it did show a substantial effect of having self-tests available at home on expected test uptake. As costs do pose a barrier to testing^10,23,26^ we recommend governments to pro-actively provide free self-tests to all its citizens—as the timely detection of COVID-19, immediate isolation and consecutive contact tracing are highly effective strategies in reducing the spread of SARS-CoV-2.

## Methods

### Study design

We did an online randomized hypothetical scenario study (vignette study) in which participants were randomised to assess four scenarios. Between subject we manipulated the testing advice (IV1; 2 levels) and the availability of self-tests (IV2; 2 levels). Corona-related symptoms were presented at random (IV3; 4 levels). We asked participants to assess their most likely behaviour on day 1 of symptoms and day 3 of unchanged symptoms (Time 1: Day of corona-related symptoms; 2 levels). And new episodes of symptoms over time were presented in additional vignettes referring to later months (Time 2: Consecutive episodes of symptoms over time; 4 levels). Symptoms (75% only one symptom and 25% more than one symptom) were based on symptom profiles found in Dutch national research1. See Table 1 for independent variables. We commissioned the data collection agency I&O Research to carry out this online vignette study, between 11 and 16th of November 2021. The Medical Review Ethics Committee of the National Institute of Public Health and the Environment (RIVM) of the Netherlands reviewed the study proposal and confirmed that the study met the ethical standards for human participation research and confirmed that further clinical approval was not needed [G&M-537]. The study was pre-registered at ClinicalTrials.gov [NCT05215483]. All methods were carried out in accordance with relevant guidelines and regulations.

**Table 1:** Variable dimensions and levels in study.

**Primary analysis**
IV1: Testing advice (between subject, randomly allocated)
1. Government guideline testing advice: “*When you have corona-related symptoms, get tested at an MHS test facility*”
2. Customised (self-)testing advice: “*When you have corona-related symptoms, get tested at an MHS test facility. If this is not possible, use self-test*”
IV2: Availability (between subject, randomly allocated)
1. Self-tests available at home
2. No self-tests available at home
**Secondary analysis**
IV3: Type of corona-related symptoms (within subject, randomly assigned per episode of symptoms)
1. One symptom: sore throat (25%)
2. One symptom: runny nose (25%)
3. One symptom: blocked nose (25%)
4. More than one symptom: Coughing and/or sneezing with a slight fever (25%)
**Timepoints measured**
Time 1: Day of corona-related symptom onset (within subject, all participants)
1. Day 1 of symptoms
2. Day 3 of symptoms (Symptoms assigned on day 1, persisted on day 3. If participants chose to visit the MHS test facility of day 1, participants were not asked about their expected behaviour on day 3 of symptoms)
Time 2: Consecutive episodes of symptoms over time (within subject, all participants)
1. Next Tuesday: mid November
2. After 4 weeks: mid December
3. After 2 weeks: beginning of January
4. After 6 weeks: end of February

### Participants

Participants were recruited by I&O Research using an online research panel representative of the Dutch population. Subjects were eligible if they were aged 18 and above, and living in the Netherlands. Invitations were sent to a selected sample based on the demographics age, gender, educational level, region and to best effort migration background. For full descriptives see Supplementary Materials (S1). Participants received credits interchangeable for online purchases for participating and informed consent was obtained from all participants.

36% of participants reported to have experienced corona-related symptoms during the past six weeks, not caused by underlying medical issues. 54.8% of these respondents say to have tested due to these symptoms, either at an MHS test facility (21.4%), with self-tests (26.3%) or both (7.1%). This is slightly higher than numbers found in Dutch representative sample studies (November 2021: 43%) during the same timeframe^29^.

21.8% of respondents report to have underlying medical issues, 18.7% have confirmed to have been tested (no self-tests) in the past 6 weeks, and 30.5% have used (at least one) self-tests in the past 6 weeks. 19.7% were experiencing corona-related symptoms during the vignette study (such as a cold, or sneezing or coughing). 18.8% (think) to have been infected with corona in the past and 91.6% are fully vaccinated for corona. Although the data collection agency aimed to provide a representative sample and was successful regarding demographics, the percentage of fully vaccinated participants (91.6%) was higher than the Dutch average in November 2021 of ~ 84%^31^.

### Interventions

Participants were automatically randomised at the start of the survey to one of four groups (advice × availability). Corona-related symptoms were randomised before the start of each scenario. Participants were asked to describe their activities on a regular Tuesday to increase immersion and informed that it is normal that people do not always follow the testing guidelines^12^. Participants were then presented with a vignette where they would wake up on a Tuesday with corona-related symptoms, their testing advice, visually presented in style of the Dutch corona campaign and text (Supplementary Materials S3), and their selected availability of self-tests in text and asked what they expected that their most likely behaviour would be: (1) no test (2) wait and see how symptoms develop (3) make an appointment with an MHS test facility or (4) use a self-test/arrange for and use a self-tests. Participants who did not choose the test facility on day 1 were presented with the same vignette referring to day 3 with unchanged symptoms, and were asked their most likely behaviour again. Participants were not aware of the hypothesis of the study.

### Study materials

We used an online questionnaire including four vignettes (see Supplementary Materials S4). Additional questions concerned demographics, previous test experience, risk profile for corona, corona-related symptoms, reasons to (not) get tested (PCR or self-tests), vaccination status, previous corona infection, own availability of self-tests, distance to nearest MHS test facility, perceived reliability self-tests, descriptive norm, risk perception, self-efficacy and response efficacy. Psychosocial constructs were measured as previous research has shown that these constructs are significant predictors for test-uptake^32^. Questions were based on the Dutch Cohort Questionnaire^33^.

### Outcomes

The primary outcomes were defined as the average strategy sensitivity over time, referring to the probability of detecting positive cases. The sensitivity of the test chosen after symptoms onset was used to convert each answer: MHS test facility on day 1 or day 3 = 1, self-test on day 1 = 0.8, self-test on day 3 = 0.7, Wait and see and No test on day 1 or day 3 = 0^7,18^. Sensitivity refers to a test’s ability to designate an individual with disease as positive. For strategy sensitivity scores on day 1 of symptoms, day 1 sensitivity scores from all four scenarios were averaged. To determine sensitivity scores by the third day of testing, the highest score was selected from day 1 or day 3 of symptoms per scenario and averaged over all four scenarios. We refer to this accumulated score as “by day 3 of symptoms”. Participants who chose to visit the MHS test facility on day 1 were not asked about their most likely behaviour again on day 3, therefore scores from day 1 were used to calculate sensitivity scores by day 3. Sensitivity scores of self-tests on day 1 are higher than day 3 as viral loads were observed to peak at the onset of symptoms and subsequently decrease^19,20^. Secondary outcomes were willingness to test sooner (day 1, self-tests or MHS test facility, averaged over four scenarios); and if respondents would test more often (by the third day of symptoms, self-tests or MHS test facility, averaged over four scenarios).

### Power

In our sample size calculations we estimated a two tailed t-test to calculate the difference between two independent groups. This resulted in a total of 1,302 participants for 95% power (α = 0.05). As three days were scheduled for data collection, we oversampled and sent out 6.053 invitations.

### Statistical methods

Sensitivity scores (day 1 and by day 3) were normally distributed based on the Skewness (day 1: − 0.27, by day 3: − 1.09) and Kurtosis (day 1: − 1.28, day 3: 0.06). Variance between groups was not equal, as shown by a significant Levene’s test for sensitivity scores on day 1 (F(3, 3266) = 6.469, *p* < 0.001), and by day 3 (F(3, 3266) = 25.10, *p* < 0.001) and Box’s test of equality of covariance matrices was significant (F(9, 119192744) = 13.594, *p* < 0.001) and therefore not all assumptions were met for parametric testing. Yet based on the large sample size and the central limit theorem, parametric tests were used to analyse the data.

As we hypothesised that changing the governmental advice on testing, and having self-tests available at home would increase the strategy sensitivity for citizens with COVID-19 symptoms on day 1 and by day 3 we used a Multivariate GLM with Bonferroni correction. Secondary outcomes, predicting that respondents would test sooner and more often, were also analysed using Multivariate GLM.

Finally, to analyse the predicted decline in strategy sensitivity over time, due to a repeated incidence of symptoms over time, we used a repeated measures ANOVA for strategy sensitivity scores on day 1 and by day 3. Analyses were done in SPSS version 28.

### Data availability

The data that support the findings of this study are not publicly available due to the European privacy regulation (GDPR) and participants of this study did not give written consent for their data to be shared publicly. Aggregated data are available from the corresponding author, C.Z., upon reasonable request.

### Supplementary Information

Supplementary Information.
