# Supplementary material for: Estimating the impact of COVID-19 self-test availability and modifications in test-strategy on overall test uptake using an experimental vignette study
Source: Sci Rep. 2024 Mar 11;14:5887. doi: 10.1038/s41598-024-54988-9 (PMC10928216; doi:10.1038/s41598-024-54988-9)
Supplement: Supplementary file 1 — Supplementary Information. [file 41598_2024_54988_MOESM1_ESM.docx]

**Supplementary material:**

**Estimating the impact of COVID-19 self-test availability and modifications in test-strategy on overall test uptake using an experimental vignette study**

**Supplementary Table 1 (S1). Participant details on demographic factors and corona related variables per condition.**

|  |  | All respondents  (N = 3270) | Government guideline testing advice (n = 1634) | Customised (self-) testing advice  (n = 1636) | No self-tests available  (n = 1648) | Self-tests available  (n = 1622) | Dutch population  (I&O research data) |
| --- | --- | --- | --- | --- | --- | --- | --- |
| Age in years |  |  |  |  |  |  |  |
|  | M (SD) | 53.3 (17.3) | 53.5 (17.4) | 53.1 (17.1) | 53.0 (17.3) | 53.6 (17.3) |  |
| Gender |  |  |  |  |  |  |  |
|  | Male | 1,609 (49.2%) | 782 (47.9%) | 827 (50.6%) | 811 (49.2%) | 798 (49.2%) | 49% |
|  | Female | 1,652 (50.8%) | 844 (51.7%) | 808 (49.4%) | 833 (50.5%) | 819 (50.5%) | 51% |
|  | X | 9 (0.3%) | 8 (0.5%) | 1 (0.1%) | 4 (0.2%) | 5 (0.3%) |  |
| Level of education* |  |  |  |  |  |  |  |
|  | Low | 712 (21.8%) | 374 (22.9%) | 338 (20.7%) | 357 (21.7%) | 355 (21.9%) | 21% |
|  | Middle | 1,245 (38.1%) | 604 (37%) | 641 (39.2%) | 637 (38.7%) | 608 (37.5%) | 40% |
|  | High | 1,313 (40.2%) | 656 (40.1%) | 657 (40.2%) | 654 (39.7%) | 659 (40.6%) | 39% |
| Ethnicity |  |  |  |  |  |  |  |
|  | Dutch | 2,601 (79.5%) | 1,312 (80.3%) | 1,289 (78.8%) | 1,297 (78.7%) | 1,304 (80.4%) | 77% |
|  | Western migration history | 408 (12.5%) | 184 (11.3%) | 224 (13.7%) | 218 (13.2%) | 190 (11.7%) | 11% |
|  | Non-Western migration history | 251 (7.7%) | 132 (8.1%) | 119 (7.3%) | 129 (7.8%) | 122 (7.5%) | 12% |
|  | Unknown | 10 (0.3%) | 6 (0.4%) | 4 (0.2%) | 4 (0.2%) | 6 (0.4%) |  |
| Household income |  |  |  |  |  |  |  |
|  | Minimum (< € 14.100) | 174 (5.3%) | 90 (5.5%) | 84 (5.1%) | 78 (4.7%) | 96 (5.9%) | 7% |
|  | Below modal (€14.100 - €29.500) | 441 (13.5%) | 227 (13.9%) | 214 (13.1%) | 239 (14.5%) | 202 (12.5%) | 14% |
|  | Almost modal (€29.500 - €36.500) | 416 (12.7%) | 208 (12.7%) | 208 (12.7%) | 204 (12.4%) | 212 (13.1%) | 12% |
|  | Modal (€36.500 - €43.500) | 537 (16.4%) | 294 (18%) | 243 (14.9%) | 260 (15.8%) | 277 (17.1%) | 16% |
|  | Between 1-2x modal (€43.500 – 73.000) | 726 (22.2%) | 352 (21.5%) | 394 (24.1%) | 361 (21.9%) | 365 (22.5%) | 22% |
|  | Twice modal (€73.000 - €87.100) | 231 (7.1%) | 109 (6.7%) | 122 (7.5%) | 123 (7.5%) | 108 (6.7%) | 7% |
|  | More than twice modal (> €87.100) | 393 (9.4%) | 147 (9%) | 162 (9.9%) | 156 (9.5%) | 153 (9.4%) | 9% |
|  | I don’t know/ Prefer not to say | 393 (12.0%)) | 188 (11.5%) | 205 (12.5%) | 202 (12.3%) | 191 (11.8%) |  |
|  | Unknown | 43 (1.3%) | 19 (1.2%) | 24 (1.5%) | 25 (1.5%) | 18 (1.1%) |  |
| Region |  |  |  |  |  |  |  |
|  | West (UT. NH. ZH) | 1,377 (42%) | 664 (40.6%) | 713 (43.6%) | 687 (41.7%) | 690 (42.5%) | 45% |
|  | Noord (GR. FR. DR) | 388 (12%) | 187 (11.4%) | 201 (12.3%) | 186 (11.3%) | 202 (12.5%) | 10% |
|  | Oost (OV. GD. FL) | 676 (21%) | 359 (22%) | 317 (19.4%) | 346 (21%) | 330 (20.3%) | 21% |
|  | Zuid (ZL. NB. LB) | 829 (25%) | 424 (25.9%) | 405 (24.8%) | 429 (26%) | 400 (24.7%) | 24% |
| Household specifics |  |  |  |  |  |  |  |
|  | I live alone | 974 (29.8%) | 491 (30%) | 483 (29.5%) | 472 (28.6%) | 502 (30.9%) |  |
|  | I love alone with children (no partner) | 112 (3.4%) | 62 (3.8%) | 50 (3.1%) | 51 (3.1%) | 61 (3.8%) |  |
|  | I live with partner without children | 1,281 (39.2%) | 647 (39.6%) | 634 (38.8%) | 671 (40.7%) | 610 (37.6%) |  |
|  | I live with partner and children | 669 (20.5%) | 319 (19.5%) | 350 (21.4%) | 331 (20.1%) | 338 (20.8%) |  |
|  | I live with my parents | 152 (4.6%) | 77 (4.7%) | 75 (4.6%) | 82 (5%) | 70 (4.3%) |  |
|  | Other | 75 (2.3%) | 34 (2.1%) | 41 (2.5%) | 39 (2.4%) | 36 (2.2%) |  |
|  | Prefer not to say | 7 (0.2%) | 4 (0.2%) | 3 (0.2%) | 2 (0.1%) | 5 (0.3%) |  |
| Vaccination status |  |  |  |  |  |  |  |
|  | Fully vaccinated | 2,994 (91.6%) | 1,493 (91.4%) | 1,501 (91.7%) | 1,512 (91.7%) | 1,482 (91.4%) |  |
|  | Not fully vaccinated | 19 (0.6%) | 9 (0.6%) | 10 (0.6%) | 12 (0.7%) | 7 (0.4%) |  |
|  | In doubt of vaccination | 58 (1.8%) | 28 (1.7%) | 30 (1.8%) | 23 (1.4%) | 35 (2.2%) |  |
|  | No vaccination | 199 (6.1%) | 104 (6.4%) | 95 (5.8%) | 101 (6.1%) | 98 (6%) |  |
| History of corona infection |  |  |  |  |  |  |  |
|  | Yes. and confirmed with a test | 366 (11.2%) | 188 (11.5%) | 178 (10.9%) | 194 (11.8%) | 172 (10.6%) |  |
|  | Yes. but not confirmed with a test | 250 (7.6%) | 132 (8.1%) | 118 (7.2%) | 129 (7.8%) | 121 (7.5%) |  |
|  | No. this is confirmed with a test | 678 (20.7%) | 333 (20.4%) | 345 (21.1%) | 330 (20%) | 348 (21.5%) |  |
|  | Probably not. but not confirmed with test | 1,624 (49.7%) | 795 (48.7%) | 829 (50.7%) | 814 (49.4%) | 810 (49.9%) |  |
|  | I don’t know | 352 (10.8%) | 186 (11.4%) | 166 (10.1%) | 181 (11%) | 171 (10.5%) |  |

*Chi-square analysis did not show any significant differences between conditions.*

**Educational level was assessed as low if participants had primary education. the first three years of middle school or vocation training level 1 (MBO-1); middle = vocational training level 2.3.4 (MBO 2.3.4) or highest levels middle school (HAVO. VWO); high = university (applied science and scientific).*

**Supplementary Table 2 (S2). Type of symptoms and choice of test.**

|  |  | No test or wait and see  % | MHS Test facility % | At least one self-test % |
| --- | --- | --- | --- | --- |
| Day 1 | More than one symptom (25%) | 22.7 | 50.2 | 27.1 |
|  | Only one symptom (75%) | 46.5 | 20.9 | 32.7 |
| By day 3 | More than one symptom (25%) | 9.7 | 71.3 | 33.0 |
|  | Only one symptom (75%) | 25.1 | 41.9 | 44.7 |
|  |  |  |  |  |
| Day 1 | More than one symptom (n=3,270) | 22.7 | 50.2 | 27.1 |
|  | Nose cold (n=3,270) | 48.9 | 17.6 | 33.5 |
|  | Runny nose (n=3,270) | 48.5 | 19.0 | 32.5 |
|  | Sore throat (n=3,270) | 42.0 | 26.0 | 32.0 |
| By day 3 | More than one symptom | 9.7 | 71.3 | 33.0 |
|  | Nose cold | 28.5 | 35.7 | 45.9 |
|  | Runny nose | 26.8 | 39.6 | 44.8 |
|  | Sore throat | 19.8 | 50.3 | 43.5 |

Percentages of selected testing behaviours by type of corona-related symptoms on day 1 and by day 3 of symptoms averaged over 4 scenario’s pooled across between-subject conditions. By day 3 also contains participants who chose to visit the MHS test facility on day 1. Percentages above 100% represent participants who expected to use a self-test on day 1. and go to the MHS test facility on day 3.

**Supplementary Material (S3). Stimuli pictures used to inform participants on advice for testing.**

1. Advice to test at an MHS test facility (Government guideline).


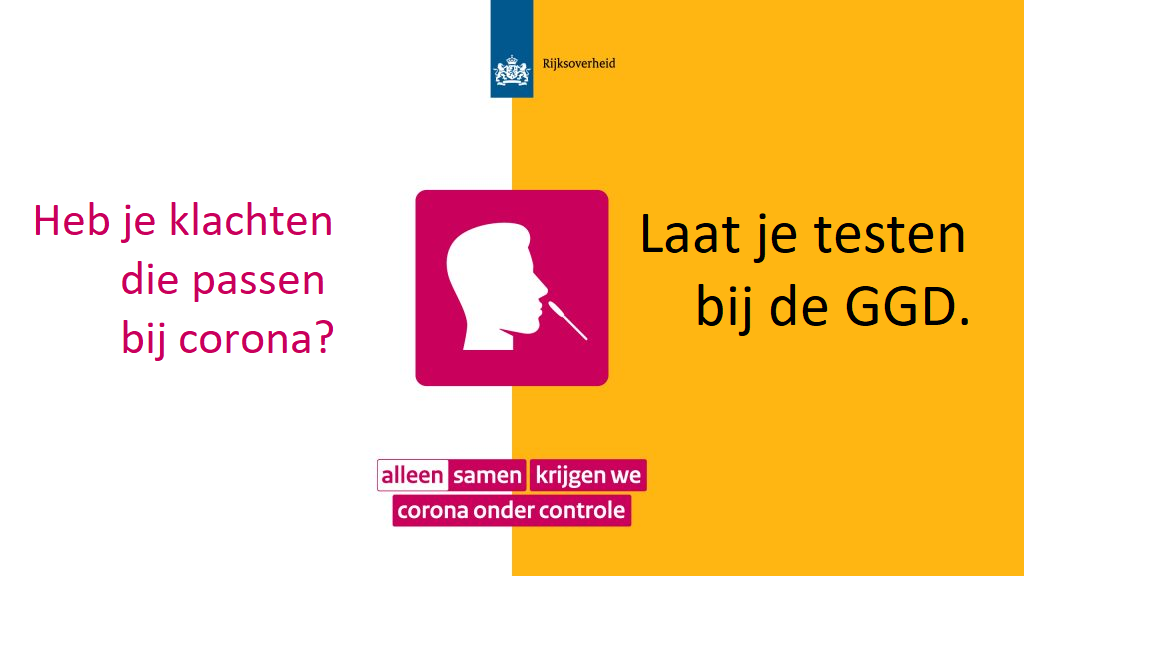


1. Advice to test at an MHS test facility. if this is not possible. use a self-test.


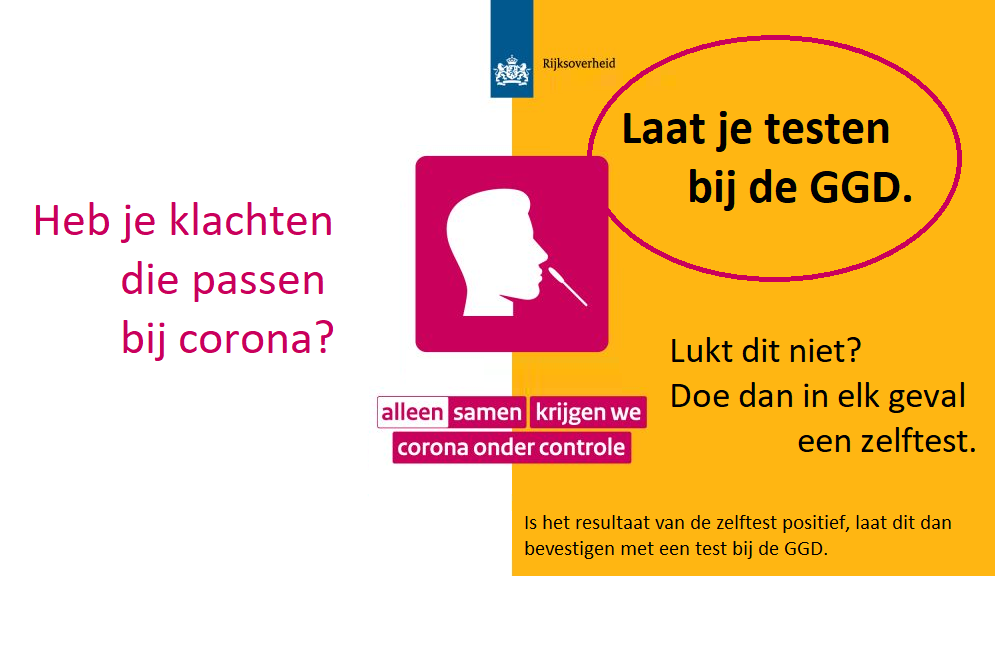


**Supplementary Material (S4). Vignette questionnaire**

**Welcome**

Thank you for participating in the survey on coronavirus and testing policies. This research is conducted on behalf of the RIVM (Dutch National Institute for Public Health and the Environment).

**Voluntary Participation**

You decide whether you want to participate in the research. Participation is entirely voluntary. You can stop filling out the questionnaire at any time. You do not have to provide a reason for doing so. This questionnaire contains questions about your health and if you have been vaccinated. These are sensitive personal data. You are not obligated to answer. By answering. you give us permission to use this data for research purposes.

All your data will be processed strictly confidentially in accordance with the guidelines of scientific research.

**Results**

Your answers will be used for scientific purposes. Anonymized data will be shared with the RIVM. The data will be securely stored to prevent unauthorized access. The research data will be kept for a minimum of 10 years after the completion of the study.

**Consent**

If you agree to participate. you consent to us processing your data for this research under the following conditions:

- Your participation in this research is entirely voluntary. and you can stop filling it out at any time without giving a reason.
- Your data will be processed solely for the purpose of the research and in accordance with the applicable privacy statement.
- The anonymized research data will be kept by the RIVM for a minimum of 10 years after the completion of the study.

We sincerely thank you in advance for your participation!

1. Do you give consent to participate in this research?

❏ Yes. I agree to the above and will fill out the questionnaire

❏ No. I do not agree to the above (you will not participate in this questionnaire)

The Netherlands has been dealing with the coronavirus for some time now. Advice and measures have been provided to the population. The purpose of this research is to gain a better understanding of how people deal with the advice and measures regarding testing for corona.

First. we would like to ask you some questions about your health and the coronavirus.

1. **Do you have one or more of the following health problems?**
   - Chronic respiratory or lung problems
   - Chronic heart conditions
   - Diabetes mellitus (diabetes)
   - Severe kidney conditions requiring dialysis or kidney transplant
   - HIV infection
   - Severe liver disease
   - Very severe obesity (BMI>40)
   - Lower resistance to infections:
     - Due to medication for autoimmune diseases
     - After organ or stem cell transplantation
     - Due to a non-functioning or missing spleen
     - In case of blood disorders
     - In case of severe immune deficiencies requiring treatment
     - Due to chemotherapy and/or radiation for cancer
     - Due to immunity-suppressing medication

□ Yes

□ No

1. **Have you been tested for coronavirus in the past 6 weeks (since October 1st). for example at the Municipal Health Service (MHS). a (commercial) testing company. or through your employer?**

*Please note: this question does not concern self-testing*

□ Yes. namely (number of tests): [OPEN]

□ No

□ I don't know

*Selection: RP has recently had a coronavirus test (not a self-test)*

1. **Why did you get tested? You can choose multiple reasons.**

Please note: this question does not concern self-testing.

*Multiple answers possible*

□ I had symptoms that could be caused by the coronavirus

□ I had done a self-test that was positive and wanted to get retested

□ I had been in contact with an infected person

□ I needed a negative test certificate to travel abroad

□ I needed a negative test certificate to return to the Netherlands from a yellow. orange. or red area

□ I got tested after returning from abroad

□ I needed a coronavirus access pass to gain entry to a cultural. social. or sporting activity or event

□ I wanted more certainty that I did not have the coronavirus

□ Other. namely: [OPEN]

1. **Have you done a self-test for coronavirus in the past 6 weeks (since October 1st)? If yes. how many times?**

Please choose the answer that best applies to your situation.

□ Yes. namely (number of tests): [OPEN]

□ No

□ I don't know

Selection: RP has recently done a self-test

1. **There are various reasons for taking a self-test. Why did you take a self-test? You can choose multiple reasons. If you have done more than one self-test in the past 6 weeks. answer this question for the most recent self-test.**

*Multiple answers possible*

□ Because I was asked by my school or employer to take a self-test

□ Because I returned from abroad

□ Because I had coronavirus-related symptoms

□ Because I was alerted that I had been in contact with an infected person

□ Because one of my household members had coronavirus-related symptoms

□ To be able to gather with more people

□ To have more certainty that I couldn't infect others during an event. gathering. or activity (such as a funeral. conference. or museum visit)

□ To not have to maintain a 1.5-meter distance from others

□ To have more certainty that I didn't have the coronavirus

□ To have more certainty that I couldn't infect others in my household

□ To have more certainty that I couldn't infect others when I visited

□ I wanted to know what it's like to take a self-test

□ Other. namely...

1. **Do you currently have one or more of the following symptoms?**

*Multiple answers possible*

□ Slight fever (temperature between 37.5 and 38 degrees Celsius)

□ Fever (temperature above 38 degrees Celsius)

□ Coughing/sneezing

□ Runny nose

□ Stuffy nose

□ Sore throat

□ Sudden loss of smell or taste

□ Difficulty breathing

□ No. I don't have any of these symptoms *

* This answer excludes the above options --> proceed to question 8.

*Selection: RP has corona related symptoms*

1. **Do you think these symptoms are caused by a condition you have had for a long time? For example. hay fever. smoker's cough. or asthma.**

□ Yes. I am very sure of this

□ Yes. probably

□ Maybe. I'm not sure

□ No. these symptoms feel different

□ No. I don't have any conditions that cause these types of symptoms

Selection: RP does not have symptoms.

1. **Have you experienced any of the following symptoms in the past 6 weeks (since October 1st)?**

*Multiple answers possible.*

□ Slight fever (temperature between 37.5 and 38 degrees Celsius)

□ Fever (temperature above 38 degrees Celsius)

□ Coughing/sneezing □ Runny nose

□ Stuffy nose □ Sore throat

□ Sudden loss of smell or taste

□ Difficulty breathing □ I don't know

□ No. I haven't experienced any of these symptoms in the past 6 weeks *

* This answer excludes the above options --> proceed to question 12.

Selection: RP has experienced symptoms in the past 6 weeks.

1. **You indicate that you have experienced one or more symptoms in the past 6 weeks (since October 1st). Do you think these symptoms were caused by a condition you have had for a long time? For example. due to hay fever. smoker's cough. or asthma.**

□ Yes. I am very sure of this

□ Yes. probably

□ Maybe. I'm not sure

□ No. these symptoms feel different

□ No. I don't have any conditions that cause these types of symptoms

Selection: RP currently has symptoms OR has experienced symptoms in the past 6 weeks (V6 or V8).

1. **Have you been tested in the past 6 weeks (since October 1st) due to these symptoms? For example. at the MHS. a (commercial) testing company. or through your employer.**

□ Yes. I have been tested (not a self-test)

□ Yes. I first did a self-test. then I got tested

□ No. I only did a self-test

□ No. I still need to make an appointment

□ No. I have made an appointment. but it hasn't taken place yet

□ No. I haven't done a coronavirus test

□ I don't know

Selection: V10 = got tested (1 or 2)

1. **Where did you get tested?**

□ Hospital or general practitioner

□ MHS Test Street

□ MHS Test Street via the priority scheme for healthcare workers and teachers

□ MHS came to me

□ At a company (on my own initiative)

□ At a company (through my employer)

□ Abroad

**We present you with a scenario in which the advice is to get a COVID-19 test. Not everyone follows this advice. for example because it doesn't fit well into their schedule or because they find a COVID-19 test unpleasant.**

**We would like you to imagine what you would do if you were in the situation shown below. It can sometimes be difficult to imagine this precisely. Therefore. try to put yourself in the moment and consider what your reaction would be. It is not important for this research to choose the 'correct answer.' but rather to reflect what you truly expect you would do.**

1. Think about what your typical Tuesday looked like over the past year. What would you normally do on this day? For example. do you go to work or school. take care of (grand)children. or have other appointments? Where do you go for these activities? Would you. for instance. do some grocery shopping in between?

You don't need to provide specific locations. but describe the places you would go (e.g.. friends' house. gym. train).

Go through a typical Tuesday and note what you would do in the morning. afternoon. and evening. and where you would go.

On Tuesday morning: _______________________________________________________

On Tuesday afternoon: _______________________________________________________

On Tuesday evening: _______________________________________________________

<page brake>

Show image: Image for testing advice is randomised between subject. One participant should only see 1 image during the whole questionnaire.


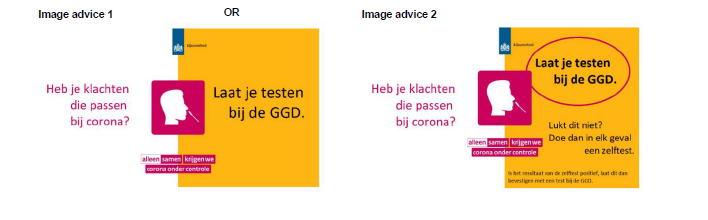


**The current government advice is “With corona-related symptoms. get tested at an MHS test facility”. [BETWEEN SUBJECT CONDITION ADVICE image 1) no further text image 2) Suppose the advice would be “With corona-related symptoms. get tested at an MHS test facility. If this is not possible. use a self-test”.**

1. Imagine. it's a regular Tuesday morning and you wake up. You notice that you have [RANDOMISE SYMPTOMS: 25% a runny nose but no cough or other symptoms / 25% a runny nose / 25% a sore throat / 25% a slight fever and need to cough and sneeze]. You have RANDOMISE AVAILABILITY [some self-tests at home / no self-tests available].

**What do you think you would do on that Tuesday? (Forced choice)**

**Check the option that is most likely for you.**

□ I decide not to get tested for this.

□ I'll wait and see how my symptoms develop and decide later.

□ I make an appointment to get tested at the MHS.

□ I do a self-test [condition: self-test available at home] / I'll do a self-test. I'll arrange to get one first [condition: no test available at home]"

*Selection: Only people who did NOT go to the MHS.*

If the answer is given "I do a self-test." provide feedback: On Tuesday. your self-test was negative.

<page brake>

**Now. imagine what you would do on a regular Thursday. What appointments do you have and where would you go? Go through a typical Thursday and think about what you would do in the morning. afternoon. and evening. and where you would go.**

<page brake>

Show image: Image for testing advice is randomised between subject. One participant should only see 1 image during the whole questionnaire.


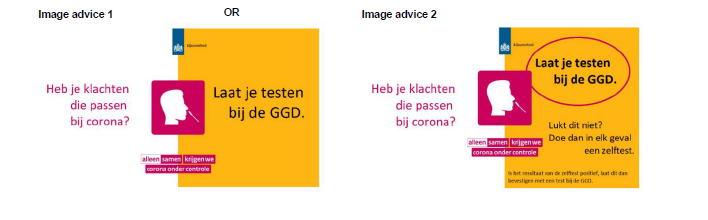
**The current government advice is “With corona-related symptoms. get tested at an MHS test facility”. [BETWEEN SUBJECT CONDITION ADVICE image 1) no further text image 2) Suppose the advice would be “With corona-related symptoms. get tested at an MHS test facility. If this is not possible. use a self-test”.**

1. It is now Thursday. two days later. You have had [Same symptom as V13] for two days now. and it has neither improved nor worsened during this time. You have [the same availability of self-tests as in V13].

**What do you think you would do on that Thursday? (Forced choice)**
**Check the option that is most likely for you.**

□ I decide not to get tested for this.

□ I'll wait and see how my symptoms develop and decide later.

□ I make an appointment to get tested at the MHS.

□ I do a self-test [condition: self-test available at home] / I'll do a self-test. I'll arrange to get one first [condition: no test available at home]"

<page brake>

If the answer given is "I take a self-test." provide feedback: Your self-test was negative. If the answer given is MHS test. provide feedback: Your MHS test was negative."

<page brake>

Show image: Image for testing advice is randomised between subject. One participant should only see 1 image during the whole questionnaire.


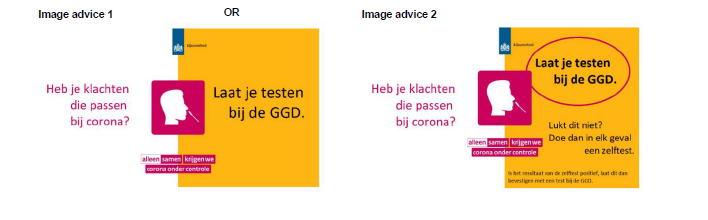
**The current government advice is “With corona-related symptoms. get tested at an MHS test facility”. [BETWEEN SUBJECT CONDITION ADVICE image 1) no further text image 2) Suppose the advice would be “With corona-related symptoms. get tested at an MHS test facility. If this is not possible. use a self-test”.**

<page brake>

Think back to what you would normally do on a Tuesday.

<page brake>

1. It is now four weeks later. and the symptoms from last time are gone. It is now mid-December 2021. Imagine. it's a Tuesday morning and you wake up. You notice that you have [RANDOMISE **SYMPTOMS**: 25% a runny nose but no cough or other symptoms / 25% a runny nose / 25% a sore throat / 25% a slight fever and need to cough and sneeze]. You have **[the same availability of self-tests as in V13].**

**What do you think you would do on that Tuesday? (Forced choice)**

**Check the option that is most likely for you.**

□ I decide not to get tested for this.

□ I'll wait and see how my symptoms develop and decide later.

□ I make an appointment to get tested at the MHS.

□ I do a self-test [condition: self-test available at home] / I'll do a self-test. I'll arrange to get one first [condition: no test available at home]"

*Selection: Only people who did NOT go to the MHS.*

If the answer is given "I do a self-test." provide feedback: On Tuesday. your self-test was negative.

<page brake>

**Think back to what you would normally do on a Thursday.**

<page brake>

Show image: Image for testing advice is randomised between subject. One participant should only see 1 image during the whole questionnaire.


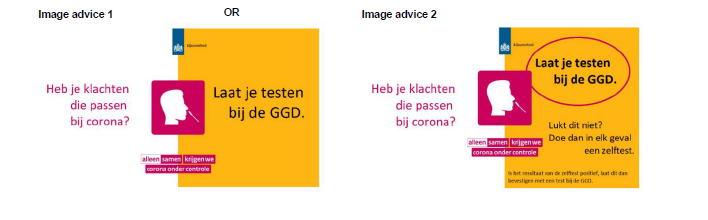


**The current government advice is “With corona-related symptoms. get tested at an MHS test facility”. [BETWEEN SUBJECT CONDITION ADVICE image 1) no further text image 2) Suppose the advice would be “With corona-related symptoms. get tested at an MHS test facility. If this is not possible. use a self-test”.**

1. **It is now Thursday. two days later in mid-December 2021. You have had [the same symptom as in V15] for two days now. and it has neither improved nor worsened during this time. You have [the same availability of self-tests as in V15].**

**What do you think you would do on that Thursday? (Forced choice)**
**Check the option that is most likely for you.**

□ I decide not to get tested for this.

□ I'll wait and see how my symptoms develop and decide later.

□ I make an appointment to get tested at the MHS.

□ I do a self-test [condition: self-test available at home] / I'll do a self-test. I'll arrange to get one first [condition: no test available at home]"

If the answer given is "I take a self-test." provide feedback: Your self-test was negative. If the answer given is MHS test. provide feedback: Your MHS test was negative.

1. **Repeat V15 for: 2 weeks after the last symptoms Early January**
2. **Repeat V16 for: 2 weeks after the last symptoms Early January**
3. **Repeat V15 for: Six weeks after the last symptoms Mid-February**
4. **Repeat V16 for: Six weeks after the last symptoms Mid-February"**

*Selection: V14 = I will not test OR I will wait and see how my symptoms develop.*

1. **In the first example. you had [symptoms from V13] for two days. You indicated that you would not test.**

**What is the reason for not getting tested? (*Multiple answers possible*)**

□ I had already taken a self-test.

□ I always have these symptoms during this period.

□ These are very mild symptoms.

□ The chance of me being infected with COVID-19 is small.

□ I prefer to wait and see how my symptoms develop.

□ I am vaccinated against COVID-19 and therefore do not think it is necessary to get tested.

□ Testing does not benefit me (it won't make me feel better).

□ I find testing very unpleasant.

□ I had already had COVID-19 and therefore do not think it is necessary to get tested.

□ I do not want to stay at home until the test result from the test centre is known.

□ The test centre is not easily accessible or too far away for me.

□ Other. namely: [OPEN]

□ I do not know *[Fixed. Exclusive]

*Selection: V13 OR V14 = I do a self-test.*

1. **In the first example. you had [symptoms from V13]. You indicated that you would take a self-test.**
   **What is the reason in this example that you prefer to choose a self-test over a test at the MHS test centre? (*Multiple answers possible*)**

□ With a self-test. I get results faster.

□ These are very mild symptoms.

□ Testing at the test centre does not benefit me (it won't make me feel better).

□ I am vaccinated against COVID-19 and therefore do not think it is necessary to get tested at the test centre.

□ It is too much hassle for me to make an appointment at the test centre.

□ I had already had COVID-19 and therefore do not think it is necessary to get tested at the test centre.

□ I did not want to stay at home until the test result was known.

□ The test centre is not easily accessible or too far away for me.

□ Other. namely: [OPEN]

□ I do not know *[Fixed. Exclusive]

*In the first example. you had [symptoms from V13]. You then indicated that you would make an appointment at the MHS.*

*Selection: V13 OR V14 = I make an appointment at the MHS.*

1. **What is the reason for you in this example to prefer a MHS test over a self-test? *(Multiple answers possible)***

□ I think a test through the MHS is more reliable than a self-test.

□ I think a test through the MHS is faster than a self-test.

□ A test through the MHS is free of charge.

□ I consider it important that my test result is included in the national infection figures.

□ I do not feel comfortable performing such a test on myself.

□ I do not feel comfortable with the idea that (commercial) self-tests are being sold for profit.

□ If I really have symptoms. I would prefer to be tested by a professional.

□ Other. namely: [OPEN]

□ I do not know *[Fixed. Exclusive]

The following questions are about your experience with the coronavirus.

1. **Vaccination against the coronavirus is voluntary; it is your choice whether you want to be vaccinated or not. Are you fully vaccinated against the coronavirus?**

□ Yes

□ Not fully yet. but the second appointment is already scheduled.

□ Not fully yet. but I still want to schedule a second appointment.

□ Not fully yet. I am unsure if I want a second vaccination.

□ Not fully yet. I do not want a second vaccination.

□ No. but the first appointment is already scheduled.

□ No. but I still want to schedule a first appointment.

□ No. I am unsure if I want a vaccination.

□ No. because I do not want to get vaccinated.

1. **Do you think you are or have been infected with the coronavirus?**

□ Yes. this has been confirmed with a test.

□ Yes. but this has not been confirmed with a test.

□ No. this has been confirmed with a test.

□ No. I do not think so. but this has not been confirmed with a test.

□ I do not know.

1. **Do you have self-tests at home?**

□ Yes. approximately _________ pieces

□ No

□ I do not know

*Selection: Self-tests at home*

1. **How did you obtain the self-test(s)? (*Multiple answers possible*)**

□ Purchased in a supermarket. drugstore. or other store

□ Purchased online

□ Received for free through school

□ Received for free through (volunteer) work

□ Received for free through a sports club or sports location

□ Received for free from the Dutch government (for example. by mail or at Schiphol Airport)

□ Received from someone I know

□ Other. namely: [OPEN]

1. **How far do you live from the nearest MHS test location? (If you are not sure. please make an estimate).**

□ 0 - 2 kilometers

□ 3 - 5 kilometers

□ 6 - 10 kilometers

□ 11 - 15 kilometers

□ 16 - 20 kilometers

□ More than 20 kilometers

1. **Imagine that you have done a COVID-19 self-test and the result is positive (meaning you are infected with the coronavirus). The advice is then to get tested again at the MHS to confirm the result. This is important to have a good overview of the infection rates in the Netherlands.**

**How likely is it that you would get tested again at the MHS after a positive self-test?**

□ I would definitely not do this

□ I would probably not do this

□ I might do this

□ I would probably do this

□ I would definitely do this

1. **If self-tests were free. I would use a self-test more often when I have COVID-19 related symptoms.**

□ Very unlikely

□ Unlikely

□ Neutral

□ Likely

□ Very likely

Selection: RP currently has symptoms OR has had symptoms in the past 6 weeks (V6 or V8)

1. **Earlier in the questionnaire. you indicated that you had experienced COVID-19 related symptoms in the past 6 weeks. The current advice is to get tested at the MHS when you have symptoms.**

**Suppose the advice had been "When you have corona-related symptoms. get tested at an MHS test facility. If this is not possible. use a self-test'" What would you have chosen?**

□ I would have gotten tested at the MHS (no self-test)

□ I would have taken a self-test

□ I would not have gotten tested

□ I do not know

1. **The current advice is "When you have corona-related symptoms. get tested at an MHS test facility." Suppose the RIVM would change the testing advice to "With symptoms. get tested at the MHS. If this is not possible. use a self-test."**

**How likely is it that you would use a self-test more often?**

□ Very unlikely

□ Unlikely

□ Neutral

□ Likely

□ Very likely

1. **To what extent do you agree or disagree with the following statement?**

**I see most people in my immediate environment get tested when they have cold-like symptoms.**

□ Completely disagree

□ Disagree

□ Neutral

□ Agree

□ Completely agree

□ I do not know

1. **I think a self-test is just as reliable as a MHS test.**

□ Completely disagree

□ Disagree

□ Neutral

□ Agree

□ Completely agree

□ I do not know

1. **How difficult or easy do you find it to get tested when you have cold-like symptoms?**

□ Very difficult

□ Difficult

□ Neutral

□ Easy

□ Very easy

□ Not applicable

1. **The current advice is "With symptoms. get tested at the MHS."**

**Suppose everyone would diligently follow this advice from the government. How much would that help in preventing the spread of the coronavirus?**

□ Does not help

□ Helps little

□ Helps a bit

□ Helps a lot

□ Helps a great deal

1. **How likely is it that you will become infected with the coronavirus in the coming months?**

□ Very unlikely

□ Unlikely

□ Neutral

□ Likely

□ Very likely

1. **How serious would it be for you if you were to contract the coronavirus?**

□ Not serious at all

□ Not serious

□ Neutral

□ Serious

□ Very serious

1. **Suppose you are infected with the coronavirus. How likely is it that you will infect others?**

□ Very unlikely

□ Unlikely

□ Neutral

□ Likely

□ Very likely

1. **How would you feel about infecting someone else with the coronavirus?**

□ Not bad at all

□ Not bad

□ Neutral

□ Bad

□ Very bad

1. **If you have any questions or comments about the questionnaire. you can mention them here.**

□ [Open text]

□ No comments

1. **How did you find it to fill out this questionnaire?**

**Can you rate the questionnaire from 1 to 10?**

A 1 stands for not pleasant at all and a 10 for very pleasant.

1-10 + do not know

**This is the end of the questionnaire. Thank you very much for your cooperation!**

**Click one more time to save your answers.**
